# The effect of bone defect size on the 3D accuracy of alveolar bone augmentation performed with additively manufactured patient-specific titanium mesh

**DOI:** 10.1186/s12903-022-02557-9

**Published:** 2022-12-01

**Authors:** Wei Yang, Dan Chen, Chao Wang, Davide Apicella, Antonio Apicella, Yuanding Huang, Linzhi Li, Lingling Zheng, Ping Ji, Lizhen Wang, Yubo Fan

**Affiliations:** 1 Stomatological Hospital of Chongqing Medical University, Chongqing, 401147 China; 2 Chongqing Municipal Key Laboratory of Oral Biomedical Engineering of Oral Higher Education Biomedical Engineering, Chongqing, 401147 China; 3 Chongqing Key Laboratory of Oral Diseases and Biomedical Sciences, Chongqing, 401147 China; 4 Key Laboratory of Biomechanics and Mechanobiology, Ministry of Education, Beijing Advanced Innovation Center for Biomedical Engineering, School of Biological Science and Medical Engineering, School of Engineering Medicine, Beihang University, Beijing, 100083 China; 5 Marrelly Health, calabrodental hospital, 88900 Crotone, Italy; 6 Advanced Materials Lab, Department of Architecture and Industrial Design, University of Campania, 81031 Aversa, Italy

**Keywords:** Alveolar bone defects, Additively manufactured patient-specific titanium mesh, Bone augmentation, Digital registration, CAD/CAM

## Abstract

**Objective:**

Additively manufactured (3D-printed) titanium meshes have been adopted in the dental field as non-resorbable membranes for guided bone regeneration (GBR) surgery. However, according to previous studies, inaccuracies between planned and created bone volume and contour are common, and many reasons have been speculated to affect its accuracy. The size of the alveolar bone defect can significantly increase patient-specific titanium mesh design and surgical difficulty. Therefore, this study aimed to analyze and investigate the effect of bone defect size on the 3D accuracy of alveolar bone augmentation performed with additively manufactured patient-specific titanium meshes.

**Methods:**

Twenty 3D-printed patient-specific titanium mesh GBR surgery cases were enrolled, in which 10 cases were minor bone defect/augmentation (the planned bone augmentation surface area is less than or equal to 150 mm^2^ or one tooth missing or two adjacent front-teeth/premolars missing) and another 10 cases were significant bone defect/augmentation (the planned bone augmentation surface area is greater than 150 mm^2^ or missing adjacent teeth are more than two (i.e. ≥ three teeth) or missing adjacent molars are ≥ two teeth). 3D digital reconstruction/superposition technology was employed to investigate the bone augmentation accuracy of 3D-printed patient-specific titanium meshes.

**Results:**

There was no significant difference in the 3D deviation distance of bone augmentation between the minor bone defect/augmentation group and the major one. The contour lines of planned-CAD models in two groups were basically consistent with the contour lines after GBR surgery, and both covered the preoperative contour lines. Moreover, the exposure rate of titanium mesh in the minor bone defect/augmentation group was slightly lower than the major one.

**Conclusion:**

It can be concluded that the size of the bone defect has no significant effect on the 3D accuracy of alveolar bone augmentation performed with the additively manufactured patient-specific titanium mesh.

## Introduction

Most dentition defects caused by trauma, congenital deformity, tooth extraction and periodontal disease require dental implants to restore bone shape, dentition integrity, and masticatory function [1, 2]. When the bone volume and contour of the alveolar bone meet specific requirements, an implant restoration can be successfully applied. Currently, autologous bone grafting, distraction osteogenesis and guided bone regeneration (GBR) are clinical routines to achieve bone augmentation [3, 4]. In dental, GBR is the most common and effective procedure for the alveolar bone volume and contour reconstruction method [5].

Titanium mesh as a non-resorbable membrane has been commonly used in dental clinical GBR surgery [6, 7]. As an effective implant device for repairing the alveolar bone defect, titanium mesh can fully maintain the osteogenic space of the alveolar bone. However, traditional titanium meshes are sheet-like and generally rigid [3, 8]. Moreover, cutting and trimming the mesh during the surgical operation is necessary; these operations prolong the operation time and rely too much on the surgeon’s clinical experience, resulting in poor control of the postoperative bone augmentation effect [9, 10]. Exposure rates of traditional titanium mesh are very high, up to 52.7%, which may be caused by the unreasonable shape design of titanium mesh or the inability to achieve the tension-free suturing [11, 12].

With the rapid development of 3D digital technology and additive manufacturing technology, Ciocca L proposed the additively manufactured (3D-printed) patient-custom-made titanium mesh for GBR [13]. The design of the patient-specific titanium mesh based on the defect site has good physical properties, space formation ability and biocompatibility [14, 15]. Compared with the traditional titanium mesh, the 3D-printed patient-specific titanium mesh has no sharp edges and corners, which can reduce the damage to the mucosa and the exposure rate of titanium mesh (0–33%). Besides, the advantages of shortening the operation time and balancing the differences in GBR surgery among surgeons with different clinical skill levels are also evident, making the bone defect re-establishment more precise and personalized [13, 16, 17]. So far, the latest literature has proved that the 3D-printed patient-specific titanium mesh can achieve excellent bone augmentation effect and meet the clinical needs [18, 19].

In the previous study, our research group analysed the systematic deviation between the planned CAD and postoperative GBR models through digital analysis of a retrospective study of 3D-printed patient-specific titanium meshes [20]. Based on the comprehensive analysis of digital registration measurement, a personalized bone augmentation using the 3D-printed patient-specific titanium mesh can be initially obtained [21, 22]. However, there is still a deviation between the planned and created bone augmentation. The reasons can be attributed to the uncertainty of the anatomical size/location of the patient’s bone defect, the reduction of anatomical landmarks, the design/manufacture of 3D-printed patient-specific titanium meshes and the offset of the implanted bone mixture [23, 24]. During the design process and surgical procedure of 3D-printed patient-specific titanium mesh, it was found that the size of the alveolar bone defect dramatically influences the design and surgical difficulty. For major bone defect/augmentation cases, many influencing factors, such as dental arrangement, alveolar bone morphology and implant position, should be considered. For minor defect/augmentation cases, the design process is more straightforward [7, 8]. Therefore, it can be speculated that the size of the alveolar bone defect may impact the 3D accuracy of bone augmentation performed with patient-specific titanium meshes.

This study aimed to investigate the 3D accuracy of alveolar bone augmentation performed with additively manufactured patient-specific titanium meshes in minor/major bone defect/augmentation cases according to the anatomical bone defect and the planned bone augmentation surface area. A set of digital data handling techniques of 20 GBR cases was enrolled, including 3D reconstruction of cone beam computed tomography (CBCT) data, 3D superimpositions of digital models and 3D comparison of bone augmentation models.

## Methods and materials

### Case selection

A total of 20 patients participated in this retrospective case study from January 2018 to December 2021. All patients had one or more missing teeth, and a 3D-printed patient-specific titanium mesh was placed in the surgical area for bone augmentation.

The inclusion criteria were as follows:

1. Adults (at least 18 years).
2. With good physical health, willing to actively cooperate with the clinical study.
3. Had undergone implant placement 6 ~ 9 months after GBR therapy with patient-specific titanium mesh in the first period.

The exclusion criteria were as follow:

1. No regular follow-up information.
2. Without complete imaging data.

According to the anatomical bone defect area and the planned bone augmentation surface area, 20 cases were divided into two groups of minor bone defect/augmentation and major bone defect/augmentation, with ten patients in each group (Table 1). This study was based on the implementation principles of the Declaration of Helsinki in 1975, revised in 2000, and approved by the ethics committee of the Stomatological Hospital of Chongqing Medical University (2018LSno.7). Patients were informed of the study protocol and signed informed consent before surgery. Distinguishing criteria for minor and major bone defect/augmentation is as follows (planned bone augmentation surface area differences are expressed as mean and standard deviation):

**Table 1:** Planned bone augmentation surface area data, expressed as min, max, mean, standard deviation

	Planned bone augmentation surface area (mm^**2**^)			
	Min (mm^2^)	Max (mm^2^)	Mean (mm^2^)	SD (mm^2^)
Minor bone defect/augmentation(*n* = 10)	56.94	122.75	88.91	45.61
Major bone defect/augmentation(*n* = 10)	180.68	504.58	88.91	96.21

**Fig. 1:**
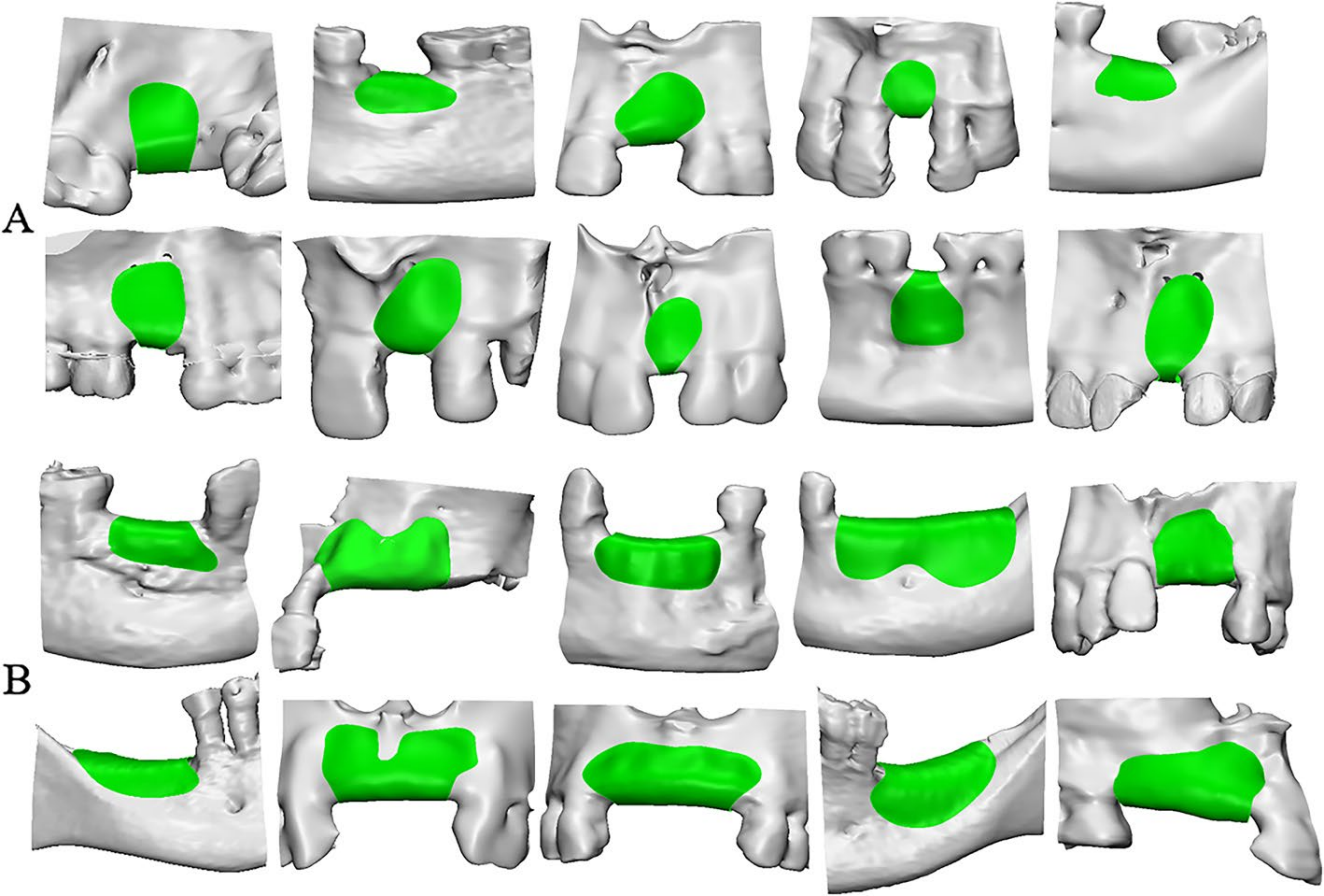
3D model of planned bone augmentation surface area in 20 patients. **A** minor bone defect/augmentation; **B** major bone defect/augmentation

Minor bone defect/augmentation (Fig. 1A):

1. The planned bone augmentation surface area is less than or equal to 150 mm^2^
2. One tooth missing or two adjacent front teeth/premolars missing

Major bone defect/augmentation (Fig. 1B):

1. Planned bone augmentation surface area is greater than 150 mm^2^
2. Missing adjacent are more than two teeth (≥3 teeth) or missing adjacent molars (≥2 teeth)

### 3D-printed patient-specific titanium mesh and guide plate

**Fig. 2:**
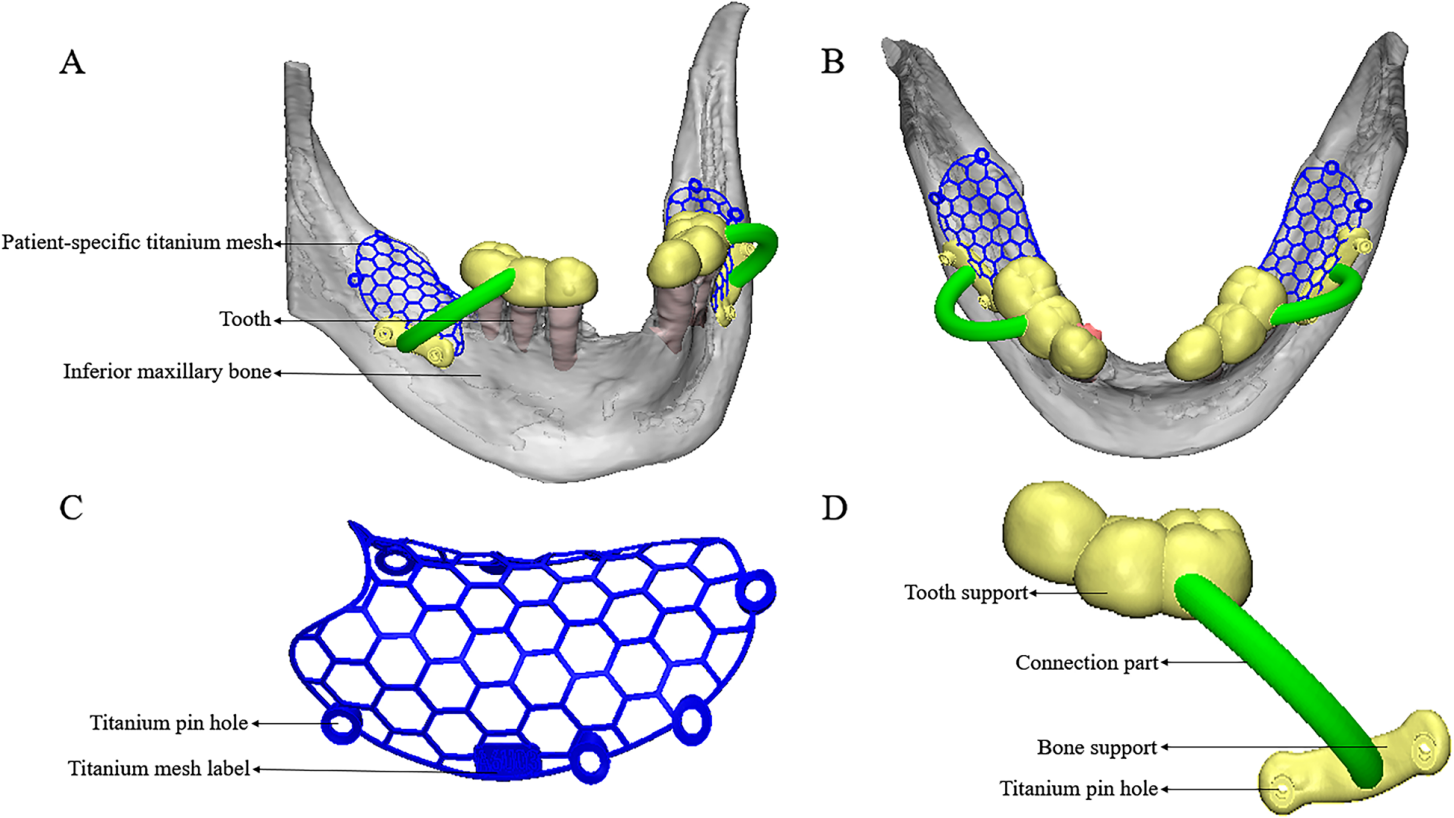
Schematic diagram of 3D-printed patient-specific titanium mesh and guide plate in bone defect (**A**) Oblique view; **B** Top view; **C** Example image of 3D-printed patient-specific titanium mesh; **D** Schematic diagram of the guide plate

The oblique and top view of 3D-printed patient-specific titanium mesh and guide plate. One titanium mesh corresponds to one guide plate, which can guide and fix the 3D-printed patient-specific titanium mesh to the pre-position (Fig. 2A, B).

3D reconstruction of the preoperative alveolar bone: digital imaging and communications in medicine (DICOM) data were obtained by preoperative CBCT (voxel size 0.4 mm;85 kV; and approximately 35 mAs; CBCT, kava, Biberach, Germany), then was imported into Mimics research software (Materialize, Leuven, Belgium) for 3D reconstruction and exported as STL file [25].

Dentition and alveolar bone virtual restoration: transfer the STL file to 3-Matic software (Materialize, Leuven, Belgium) to simulate implant placement and tooth crown restoration to determine planned bone augmentation.

The design of 3D-printed patient-specific titanium mesh based on bone contour (Fig. 2C): firstly, the thickness of the titanium mesh is 0.3 mm, and the inner diameter of the titanium pin holes is 2 mm. Secondly, the edge of the titanium mesh should be kept at least 2 mm away from vital anatomical structures such as teeth, nerves and blood vessels. Finally, 2 ~ 6 titanium pin holes with a 2 mm diameter should be carefully designed.

The design of guide plate (Fig. 2D): the guide plate structure was designed according to the titanium pin holes of the titanium mesh and the dentition in 3-Matic software (Materialize, Leuven, Belgium) to facilitate the positioning of the 3D-printed patient-specific titanium mesh during GBR.

The manufacturing of 3D-printed patient-specific titanium mesh: medical-grade titanium alloy powder (Dentarum Ti6Al4V, Germany) was applied to fabricate the 3D-printed patient-specific titanium mesh.

The manufacturing of the guide plate: the photosensitive resin material was used to 3D-print the guide plate by digital light processing technology [26].

### Surgical procedure

**Fig. 3:**
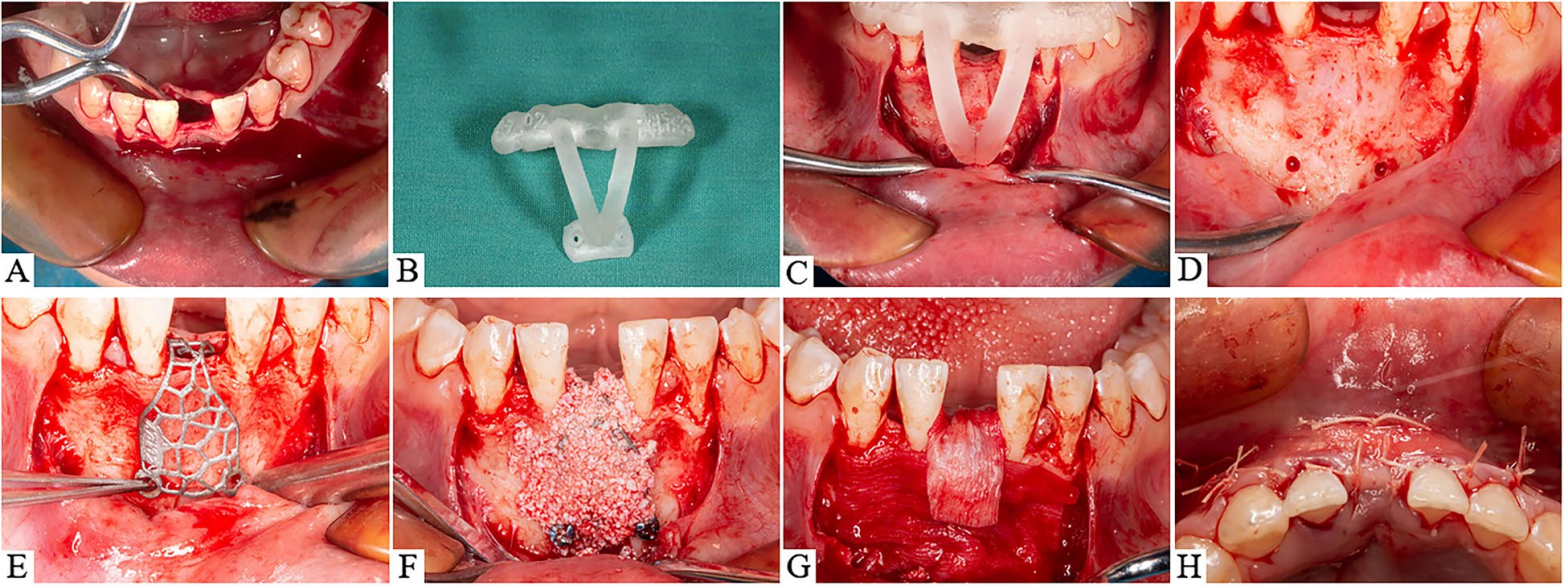
Surgical implantation procedure of 3D-printed patient-made titanium mesh under guide plate positioning; **A** Bone defect area was dissected; **B** Real picture of guide plate; **C** Guide plate in place; **D** The display of titanium pin holes; **E** Titanium pins fixed 3D-printed patient-specific titanium mesh; **F** Filling with bone graft; **G** Resorbable membrane covers bone graft; **H** Periosteal flap aligned and sutured

Before the operation, 7 ~ 10 ml of blood was drawn from the patient’s anterior elbow vein to make injectable platelets-rich fibrin (i-PRF)*.* A compound gargle solution of chlorhexidine was gargled for 1 min, disinfected the operation area with iodophor three times and then used 4% of articaine solution for local anaesthesia. Firstly, a “linear” incision on the alveolar ridge in the operation area was made. Then, it was expanded 1 ~ 2 tooth positions in the non-operation area on both sides, added the vertical incision to avoid damaging the gingival papilla, and opened the mucoperiosteal flap (Fig. 3A). Autogenous bone particles (chin, external oblique line of mandible) were collected with a ring bone drill. Several tiny round holes were drilled in the non-bone-defect area to provide blood supply and collect bone particles. An equal mixture (1:1) of autologous bone particles and deproteinized bovine bone mineral (Bio-Oss, Geistlich Pharma AG, Wolhusen, Switzerland) was mixed as a whole with i-PRF for 10 minutes until it became into the “sticky” bone graft. Checked the guide plate (Fig. 3B). Drilled on the titanium pin holes of the guide plate after the guide plate was in place (Fig. 3C). Titanium pin holes of 3D printed patient-specific titanium mesh had been drilled (Fig. 3D). The procedure first involves locating the titanium mesh at the alveolar bone defect and then implanting two or more titanium pins (Fig. 3E). The prepared bone graft was placed into the bone defect space below and above the titanium mesh (Fig. 3F). After bone graft implantation, the resorbable membrane was covered between the 3D-printed patient-specific titanium mesh and mucosal soft tissue (Fig. 3G). Lastly, all flaps were closed with a modified horizontal or vertical mattress suture to reduce tension for better fixation of the absorbable membrane and titanium mesh (Fig. 3H). Patients were called back for following visits every month after the surgery to investigate the healing and post-surgical complication. CBCT scan and subsequent surgery were performed after 6 to 9 months of healing.

### Acquisition and registration of 3D models

Mimics Research software (Materialize, Leuven, Belgium) was used to extract a 3D model of the maxilla/mandible where the bone defect was located in pre-GBR CBCT of all patients and save it as a “pre-GBR model” STL file.

The pre-GBR model of the patient was imported into 3-Matic software (Materialize, Leuven, Belgium) for planned bone augmentation design and then stored as a whole “planned-CAD model” STL file.

The target alveolar bone model was extracted from CBCT 6 ~ 9 months after GBR by Mimics Research software (Materialize, Leuven, Belgium) and stored as a “post-GBR model” STL file.

Three 3D models (STL files) of the same patient were imported into Geomagic software (Geomagic, NorthCarolina) for digital registration, and some anatomical landmarks for about the distance of 2 ~ 3 teeth on both sides of each model were selected. After processing of smooth network, three 3D model files (STL files) are exported, and 20 groups of registered 3D models are obtained.

### Superimposition of registered 3D models

A superimposition technique was used to compare the difference between the planned and created bone augmentation in minor or major bone defect/augmentation groups. Each case’s planned-CAD model and post-GBR model were imported into 3-Matic software (Materialize, Leuven, Belgium) and superimposed to analyze the difference of the bone augmentation between planned and created, and mean ± SD (mm) was used to describe the result.

### Models bone outline display

Pre-GBR model, planned-CAD model, and post-GBR model of all patients were imported into Mimics Research software (Materialize, Leuven, Belgium). Then, the bone profiles of the three models have been marked on the cross-sectional grey background CBCT views as yellow, orange, and green lines, respectively. Yellow line: bony profile of the pre-GBR models; Orange line: bone profile of the planned-CAD models; green line: bone profile of the post-GBR models.

### Statistical analysis of the difference in bone augmentation between minor and major bone defect/augmentation

GraphPad Prism 9.3.1 (GraphPad Software) was performed for all statistical analyses. Descriptive statistical analysis was used to show the superposition results of the three-dimensional models. Two groups of patients were based on an unpaired t-test to assess the difference between the planned and created bone augmentation in minor or major bone defect/augmentation groups (minimum, maximum, mean and the analysis results with standard deviations are shown in histograms). The means, SDs and 95% confidence intervals (CIs) were calculated to determine the accuracy of bone augmentation.

## Results

### Patient profile

This study included 8 females and 12 males in the 20 patients. The mean age of patients (21–56 years) was 35.7 years. Five cases were smokers with periodontal disease. There were only five cases of the thin gingival type, while the rest were the thick gingival type. Seven of the 20 bone defects were in the maxilla and 13 in the mandible. Tooth loss ranged from 1 to 5 teeth (average two teeth). Of the 20 patients, 12 had mixed alveolar bone defects, 6 had vertical bone defects, and 2 had horizontal bone defects. The characteristics of all patients are summarized in Table 2.

**Table 2:** Characteristics of enrolled patients

Mean age (range)	35.7 years(21–56)
**Female/Male (*n*** **= 20)**	8/12
**Smoker**	5
**Periodontal disease**	5
**Gingival morphotype (thick/thin)**	5/15
**Maxillary/mandibular cases**	7/13
**Mean missing teeth sites (range)**	2 Teeth (1–5)
**Mixed/vertical/horizontal bone defects**	12/6/2

### Clinical outcomes

After 1 ~ 9 months of follow-up after GBR with 3D-printed patient-specific titanium meshes, soft tissue healing was successful in 17 of 20 patients (85%), and dehiscence occurred in 3 patients (15%). One case occurred in the minor bone defect/augmentation group, and the palatal titanium mesh was exposed 6 months after the surgery. Two cases occurred in the major bone defect/augmentation group, one had exposed alveolar crest 3 months after surgery, and the other had titanium mesh exposed on both the palatal side and alveolar crest 5 months after surgery. No signs of infection were found in any of the 3 cases, and no special treatment was given except for twice-daily rinsing with a 0.12% chlorhexidine mouthwash until soft tissue healed.

### Superimposition and contour deviation

**Fig. 4:**
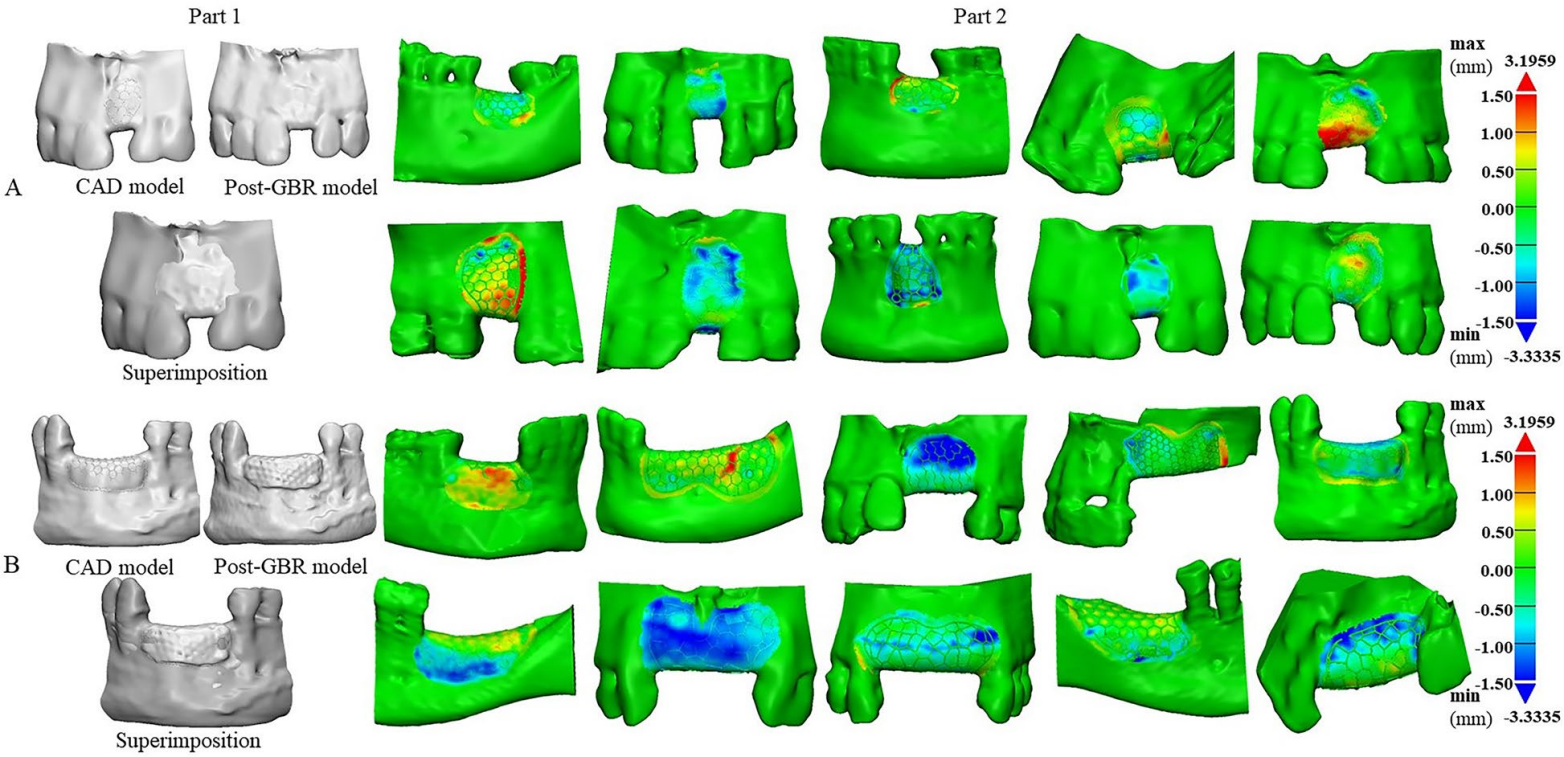
Superimposition of the digital models, the blue surface shows the negative distance between the superimposed models, and the red surface indicates the positive spread between them. **A** Analysis schematic cloud map of planned-CAD model and post-GBR model in minor bone defect/augmentation group; **B** Analysis schematic cloud map of planned-CAD model and post-GBR model in major bone defect/augmentation group

The deviations among the outcome of color mapping in each group’s planned-CAD model and post-GBR model were calculated by Materialize 3-Matic. More blue-color mapping represented the created bone augmentation more than the planned bone augmentation. Minimum values (the negative distances) represented the post-GBR model over the planned-CAD model. In contrast, maximum values (the positive distances) represented the post-GBR model below the planned-CAD model. Automatic analysis showed that the minimum deviations between post-GBR and planned-CAD models reached − 3.33 mm (Fig. 4A, B).

Descriptive analysis showed that in the 10 cases of minor bone defect/augmentation, the minimum deviation between post-GBR and planned-CAD models was − 2.02 ± 0.42 mm, while the maximum deviation was 1.72 ± 0.74 mm, and the average deviation was − 0.22 ± 0.46 mm (Table 3A). The minimum divergence between post-GBR and planned-CAD models in the 10 major bone defect/augmentation was − 2.72 ± 0.73 mm, the maximum was 1.46 ± 0.75 mm, and the mean deviation was − 0.42 ± 0.52 mm (Table 3B).

**Table 3:** Using Materialize 3-Matic software descriptive analysis of the divergences between planned-CAD models and post-GBR models in 3D-printed patient-specific titanium meshes, expressed as mean and standard deviation of deviation distances (A) minor bone defect/augmentation;(B) major bone defect/augmentation

**The divergence between CAD designing models and post-GBR models in minor bone defect/augmentation**				
	Min (negative)	Max (positive)	Mean absolute value	SD
Mean (mm)	−2.02	1.72	−0.22	0.53
SD (mm)	0.42	0.74	0.46	0.16
**B The divergence between CAD designing models and post-GBR models in major bone defect/augmentation**				
	Min (negative)	Max (positive)	Mean absolute value	SD
Mean (mm)	−2.18	1.46	−0.42	0.63
SD (mm)	0.73	0.75	0.52	0.15

**Fig. 5:**
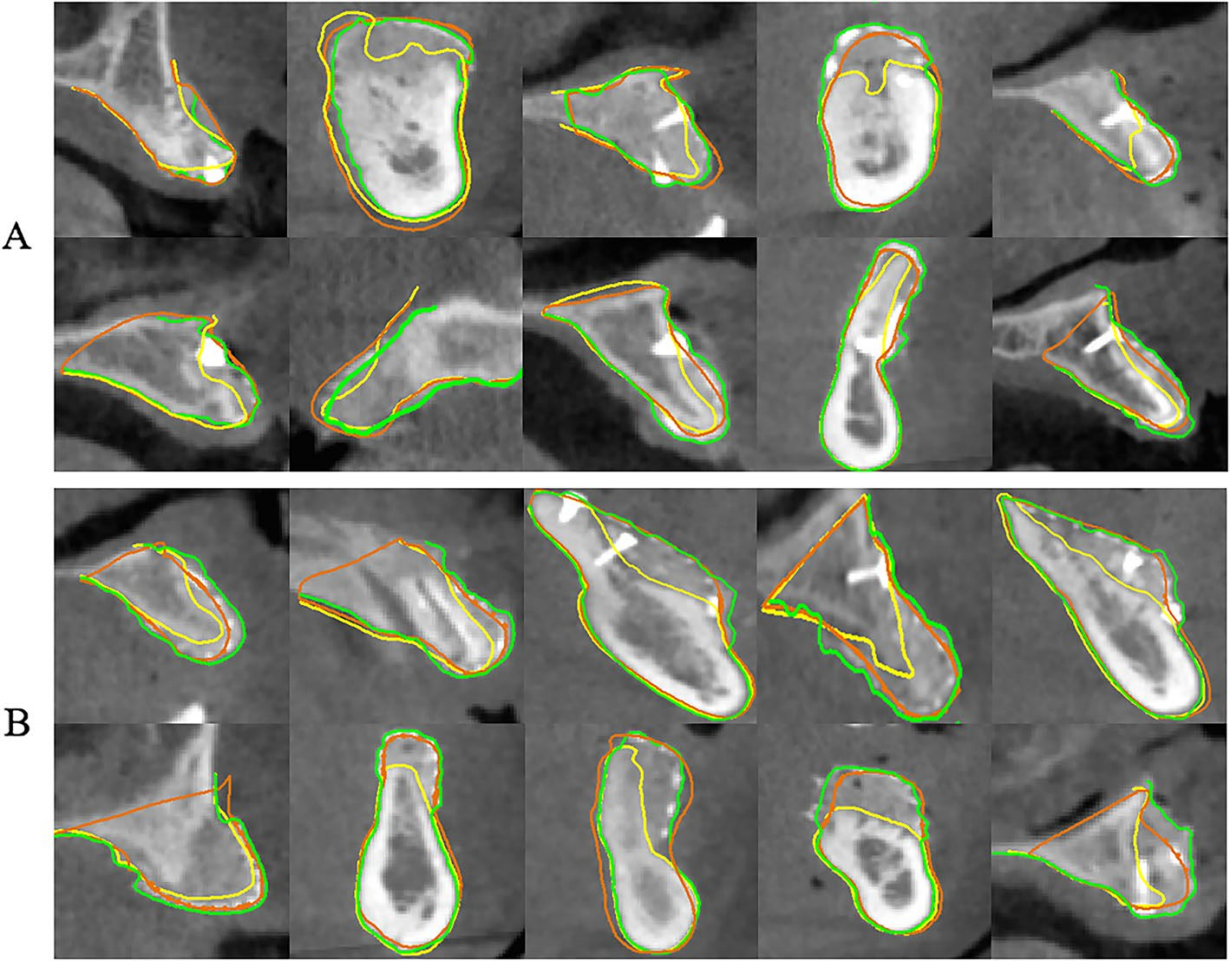
Schematic diagram of bone external profiles of the pre-GBR model, planned-CAD model and post-GBR model for each patient. Yellow line: bone profile of pre-GBR model; Orange line: bone profile of planned-CAD model; green line: bone profile of post-GBR model; **A** Schematic diagram of the bone profile lines of the digital model in the minor bone defect/augmentation group; **B** Schematic diagram of the bone profile lines of the digital model in the major bone defect/augmentation group

Schematic diagrams of bone contours of the pre-GBR model (yellow lines), planned-CAD model (orange lines) and post-GBR model (green lines) for each patient are reported. Whether the minor bone defect/augmentation (Fig. 5A) or the major bone defect/augmentation (Fig. 5B), the pre-GBR model was below the planned CAD and post-GBR models in every case.

### Bone volumetric difference between major and minor bone defect/augmentation

Through digital software analysis, recorded the minimum, maximum, and average deviation distance of planned-CAD models and post-GBR models of two bone defect groups, six sets of data in total. The minor bone defect/augmentation group and the major bone defect/augmentation group were analyzed by unpaired t-test in the minimum, maximum, or average deviation distance. The analysis data showed that 95% confidence interval of the minimum deviation distance was − 0.74 mm to 0.44 mm, the difference (Mean ± SD) was − 0.55 ± 0.28 mm, and *P*-value was 0.5922 (df = 18, *p* < 0.05); 95% confidence interval of the maximum deviation distance is − 0.99 mm to 0.48 mm, and the difference (Mean ± SD) is − 0.26 ± 0.35 mm, *P*-value 0.4677 (df = 18, *p* < 0.05); 95% confidence interval of mean distance deviation is − 0.68 mm to 0.29 mm, the difference (Mean ± SD) is − 0.20 ± 0.23 mm, *P*-value 0.4032 (df = 18, *p* < 0.05) (Table 4).

**Table 4:** Unpaired t-tests showed differences in the minimum deviation distance, the maximum deviation distance, the mean deviation distance and the standard deviation value between planned-CAD models bone augmentation vs post-GBR bone augmentation in minor bone defect/augmentation or major bone defect/augmentation

Planned-CAD model vs post-GBR bone augmentation in minor bone defect/augmentation vs Planned-CAD model vs post-GBR bone augmentation in major bone defect/augmentation			
	Difference	95%CI	*P*-value
	(Mean + SD)	(difference)	
Min value (negative)	−0.55 ± 0.28	−0.74 to 0.44	0.5922
Max value (positive)	−0.26 ± 0.35	−0.99 to 0.48	0.4677
Mean absolute value	−0.20 ± 0.23	−0.68 to 0.29	0.4032
Standard deviation	0.09 ± 0.07	−0.06 to 0.24	0.2252

**Fig. 6:**
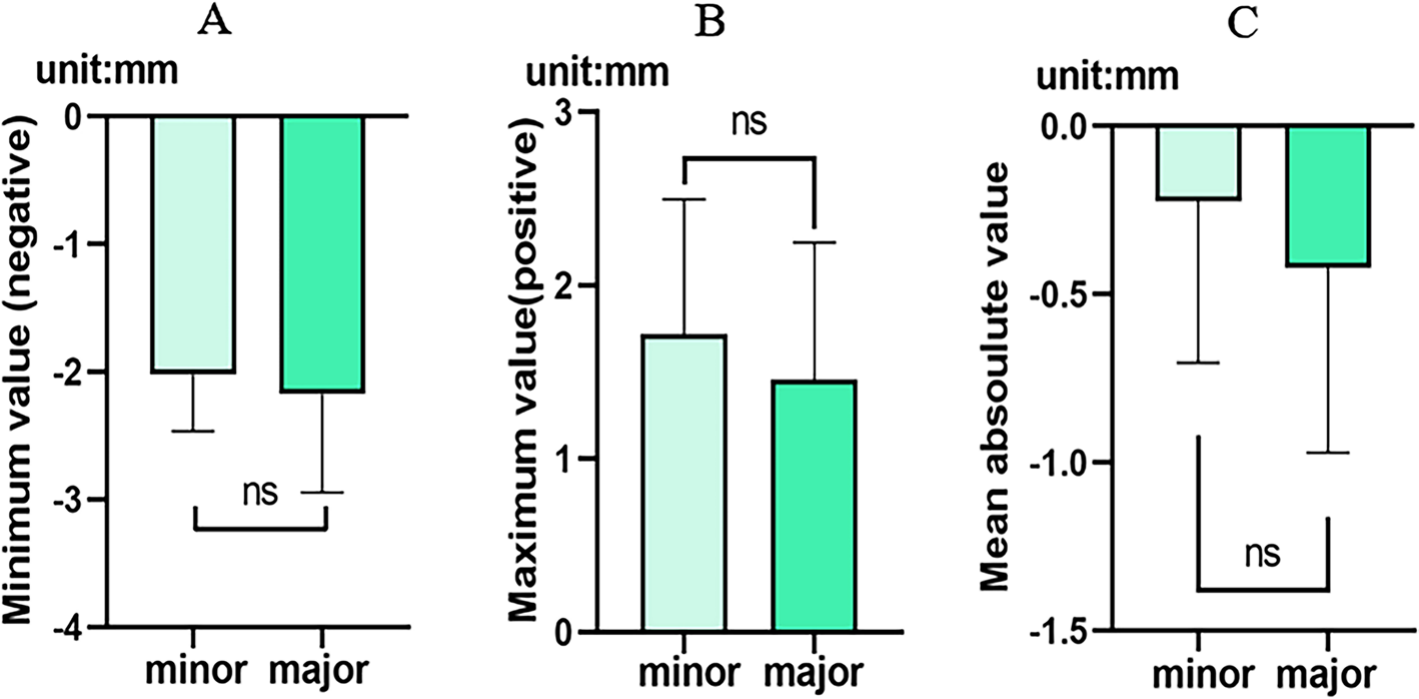
Histograms of the unpaired t-test, **A** The minimum deviation distance of the major bone defect/augmentation was smaller than that of minor bone defect/augmentation, even if the differences were not statistically significant; **B** The maximum deviation distance of major bone defect/augmentation was smaller than that of minor bone defect/augmentation, even if the differences were not statistically significant; **C** The mean deviation distance of major bone defect/augmentation was more extensive than that of minor bone defect/augmentation, even if the differences were not statistically significant

After the unpaired t-test, the histograms showed that the minimum deviation distance of major bone defect/augmentation was slightly smaller than that of minor bone defect/augmentation. Still, the difference was not statistically significant (Fig. 6A). The maximum deviation distance for major bone defect/augmentation in the maximum deviation distance was slightly smaller than that for minor bone defect/augmentation, while the difference was not statistically significant (Fig. 6B). The mean deviation distance of major bone defect/augmentation is smaller than that of minor bone defect/augmentation, but the difference is not statistically significant (Fig. 6C). The value of the maximum deviation distance of the major bone defect/augmentation is smaller than that of the minor bone defect/augmentation. In contrast, the minimum deviation distance and the mean deviation distance of the major bone defect/augmentation are greater than those of the minor bone defect/augmentation, indicating that the created bone augmentation of the post-GBR models in the major bone defect/augmentation is more significant than that of the planned-CAD models.

## Discussion

GBR is the gold standard for bone regeneration in bone grafting methods, and the biological basis of this technique focuses on the “PASS” principles: primary closure, angiogenesis, space maintenance, and clot stabilization [27]. The use of barrier membranes to stabilize bone graft materials’ space promotes osteoblasts’ migration and proliferation. It prevents soft tissue colonisation in bone defects, which is the key to GBR surgery [5, 28, 29]. However, the collagen barrier membranes lack sufficient mechanical properties to maintain the osteogenic space effectively. The traditional titanium mesh lacks enough flexibility and is difficult to shape and trim. Therefore, the additively manufactured patient-specific titanium mesh has gradually attracted the attention of dental clinicians.

During the GBR surgery performed with the additively manufactured patient-specific titanium mesh, the 3D accuracy of alveolar bone augmentation is affected by many factors. For example, although the titanium mesh is carefully designed, the multiple smooth superpositions of the digital 3D model during the digital reconstruction process will also lead to inconsistency of the bone contour. The remaining anatomical landmarks of the model with alveolar bone defects may be reduced after multiple smoothing and superposition [20, 21, 30]. Therefore, installation and retention during clinical operations still depend on the surgeon’s judgment of the anatomical position. Furthermore, placing 3D-printed patient-specific titanium meshes at the defect site, with and without bone graft, is very different [31, 32]. Part of the anatomical landmarks covered by the bone mixture can hinder the positioning of the titanium mesh in the correct position to a certain extent, resulting in the movement or misalignment of titanium mesh [33]. At the same time, it is also possible that the titanium pinhole is misaligned due to the insufficient anatomical location of exposure during the operation [34]. In addition, according to the images provided in this study, the differences between the planned volume and the actual results also come from either displacement of graft particles, over augmentation and graft remodeling during the healing period.

Based on the experience of titanium mesh design and GBR surgical procedures, we initially hypothesized that the 3D accuracy of bone augmentation should be affected by the size of an alveolar bone defect. Too much loss of anatomical landmarks and too more the planned bone augmentation surface area in the design of the titanium mesh in the major bone defect/augmentation cases; as a result, the design of titanium mesh does not conform to the anatomical characteristics of the alveolar bone to wholly restore alveolar bone defects, making the area of bone augmentation excessive or insufficient [35, 36]. On the contrary, there are more prominent anatomical landmarks and less surface area of planned bone augmentation in minor bone defect/augmentation cases, allowing the titanium mesh design to be as close as possible to the anatomical alveolar bone without gaps [37, 38]. However, the results of this study do not support this hypothesis. There was no significant difference in the 3D deviation distance of bone augmentation between the minor bone defect/augmentation group and the major one. The contour lines of planned-CAD models in two groups were basically consistent with the contour lines after GBR surgery, and both covered the preoperative contour lines. The exposure rate of titanium mesh in the minor bone defect/augmentation group was slightly lower than the major one, which could be attributed to the smaller defect area, less tension and easier mucosal healing.

The possible reasons are as follows: First, the fabrication accuracy of titanium mesh based on additive manufacturing is very high. Although major bone defect/augmentation increases the design difficulty during the design process, once the 3D design model is completed, the high-precision laser additive manufacturing process can ensure the precision machining of titanium mesh. Second, 3D-printed titanium mesh has excellent structural stiffness. Unlike traditional titanium mesh, the stiffness of 3D-printed titanium mesh is large enough to ensure that only small deformation occurs. Third, the patient-specific titanium mesh can ensure sufficient positioning accuracy due to the meticulous digital design preceding the surgical operation.

Of course, the limitation of the retrospective study should be admitted herein. In addition to the size of the bone defect, such as living habits, periodontitis, and other factors are not controllable, which will also affect postoperative bone augmentation. Therefore, more clinical studies are needed in the future to illustrate further the impact of different sizes of bone defects on the accuracy of bone augmentation performed with the additively manufactured patient-specific titanium mesh.

## Conclusion

It can be concluded that the 3D accuracy of bone augmentation is not significantly affected by the size of the alveolar bone defect according to the comparison between the minor and major bone defect/augmentation groups. Furthermore, it can be inferred with caution that inaccuracies between planned and created bone volumes and contours may not be attributed to various anatomical locations of the alveolar bone defect.

## Data Availability

The datasets generated and analyzed during the current study are not publicly available due to the subject matter specialization but are available from the corresponding author upon reasonable request.

## Abbreviations

3D: 3-Dimensions
GBR: Guided bone regeneration
CAD/CAM: Computer-aided design/Computer-aided manufacturing
CBCT: Cone beam computer tomography
DICOM: Digital imaging and communications in medicine
i-PRF: injectable-Platelets rich fibrin
Min: Minimum
Max: Maximum
CI: Confidence interval
SD: Standard deviation
